# Chemo-immunotherapy induces tumor regression in a mouse model of spontaneous mammary carcinogenesis

**DOI:** 10.18632/oncotarget.10880

**Published:** 2016-07-28

**Authors:** Eleonora Aricò, Paola Sestili, Giulia Carpinelli, Rossella Canese, Serena Cecchetti, Giovanna Schiavoni, Maria Teresa D'Urso, Filippo Belardelli, Enrico Proietti

**Affiliations:** ^1^ Department of Haematology, Oncology and Molecular Medicine, Istituto Superiore di Sanità, Rome, Italy; ^2^ Department of Cell Biology and Neurosciences, Istituto Superiore di Sanità, Rome, Italy

**Keywords:** chemotherapy, immunotherapy, cancer, ACT, HER-2 mice

## Abstract

Tumor-specific immune tolerance represents an obstacle for the development of effective anti-tumor immune responses through cancer vaccines. We here evaluated the efficacy of chemo-immunotherapy in breaking tumor-specific immune tolerance in an almost incurable mouse model of spontaneous carcinogenesis.

Transgenic HER-2/neu mice bearing large mammary tumors received the adoptive transfer of splenocytes and serum isolated from immune donors, with or without pre-conditioning with cyclophosphamide. Treatment efficacy was assessed by monitoring tumor growth by manual inspection and by magnetic resonance imaging. The same chemo-immunotherapy protocol was tested on tumor-free HER-2/neu mice, to evaluate the effects on tumor emergence.

Our data show that chemo-immunotherapy hampered carcinogenesis and caused the regression of large mammary tumor lesions in tumor-bearing HER-2/neu mice. The complete eradication of a significant number of tumor lesions occurred only in mice receiving cyclophosphamide shortly before immunotherapy, and was associated with increased serum anti HER-2/p185 antibodies and tumor leukocyte infiltration. The same protocol significantly delayed the appearance of mammary tumors when administered to tumor-free HER-2/neu mice, indicating that this chemo-immunotherapy approach acted through the elicitation of an effective anti-tumor immune response. Overall, our data support the immune-modulatory role of chemotherapy in overcoming cancer immune tolerance when administered at lymphodepleting non-myeloablative doses shortly before transfer of antigen-specific immune cells and immunoglobulins. These findings open new perspectives on combining immune-modulatory chemotherapy and immunotherapy to overcome immune tolerance in cancer patients.

## INTRODUCTION

The recent success of some novel immunomodulatory regimen shed new light on immunotherapy as a promising approach for the treatment of cancer and chronic diseases. Since the immune response spontaneously raising against nascent tumors is often inadequate, adoptive cell transfer (ACT) can be used as a mean to selectively amplify an antitumor T-cell response that can be potentially effective *in vivo*, as well as a way to overcome the limitations of vaccine-based immunization in patients that are often immunocompromised or tolerant to tumor antigens [1]. Of note, the rate of immunotherapy success in the clinic is relatively low, and the lack of correlation between immune response elicited and clinical efficacy achieved has been often reported [2]. Besides the unfortunate balance between the rapid tumor growth and the slow development of a tumor-specific immune response, it has to be underlined that cancer cells set up several immune evasion strategies, by creating an immunosuppressive microenvironment through the recruitment of regulatory T cells (Tregs) and other immune suppressors, or by selecting less immunogenic tumor variants [3, 4].

Different strategies have been employed in clinical and pre-clinical studies to improve the anti-tumor immune response, including infusion of tumor-infiltrating lymphocytes (TILs), lymphokine-activated killer cells (LAKs), vaccine-primed lymphocytes, T cells genetically engineered to express tumor-specific antigen receptors, and T cells with chimeric antigen receptors (CARs) [1]. The most promising results have been accomplished in clinics by immunotherapy approaches specifically aimed at breaking tumor-induced tolerance, in particular by passive immunotherapy based on T-cells checkpoints restraint [5].

In all settings, preconditioning with chemotherapy represents an important component of ACT, and a substantial increase in cell persistence and clinical responses was achieved in patients receiving a lymphodepleting preparative regimen [1].

The possibility to obtain regression of experimental tumors by combining a chemotherapeutic agent, such as cyclophosphamide (CTX), with adoptive immunotherapy was firstly reported in the early eighties, and represented an innovation in the use of chemotherapy in cancer [6, 7]. Later, the combination of CTX and adoptive immunotherapy proved to be effective in several animal models in reverting tumor-induced tolerance for cancer treatment [8], and was more recently translated to clinical settings [1]. Our group and others demonstrated that the rationale of combining cytotoxic chemotherapy with immunotherapy relies on: i) the elimination of Tregs caused by chemotherapy-induced lymphodepletion [9, 10], ii) the immunogenic signals released by dying cancer cells [11], and iii) the homeostatic proliferation of immune cell pools occurring immediately after chemotherapy discontinuance and correlating with immune function recovery [12].

Female mice transgenic for the activated rat HER-2 oncogene (Neu and ErbB-2 in humans), under the control of the mouse mammary tumor virus promoter, inexorably develop invasive carcinomas in all their mammary glands by the age of six months [13]. It has been reported that the rat HER-2 protein, differing by <6% of amino acid residues from the mouse homologue, can be immunogenic in wild type mice [14]. However, the transgene expression in the thymus during HER-2/neu mice development causes the negative selection of reactive cells, rendering these mice tolerant against the transforming oncogene. Therefore, HER-2/neu mice represent a valuable model of immune tolerance against the spontaneous development of mammary tumors and, as such, they have been extensively used to study the biology of breast cancer as well as to test the efficacy of therapeutic or prophylactic vaccination strategies [15]. Interestingly, HER-2/neu tolerized mice were also used as a model to prove that CTX, doxorubicin, docetaxel and paclitaxel augmented the activity of tumor vaccines [16]. Although some cancer vaccine approaches have limited the growth of autochthonous or transplanted tumors in mice [17] and the delay of spontaneous tumors development has been sometimes achieved in HER-2/neu transgenic mice [18], the cure of established spontaneous Her-2/Neu tumors have been only obtained when very early lesions were frequently treated over several cycles [19–21].

In this study, we tested the combination of non myeloablative doses of CTX with the adoptive transfer of tumor-immune cells and immunoglobulins as antitumor vaccine and obtained, for the first time to the best of our knowledge, the complete regression of large established tumors spontaneously developed in HER-2/neu transgenic mice. These observations shed new light on the possibility to overcome immune tolerance in cancer patients by combining immune-modulatory chemotherapy and immunotherapy.

## RESULTS

### Immunization strategies against a HER-2 expressing transplantable tumor

A clonal tumor cell line, named 676-1-25, was isolated from a mammary tumor developed in a HER-2/neu transgenic mouse and stabilized *in vitro* (see Supplementary Materials and Methods). The 676-1-25 cells expressed HER-2/neu protein p185, replicated *in vitro* and developed self-limiting tumor masses in a dose-dependent manner when injected s.c. in syngeneic non-transgenic mice (Supplementary Figure S1).

**Figure 1:**
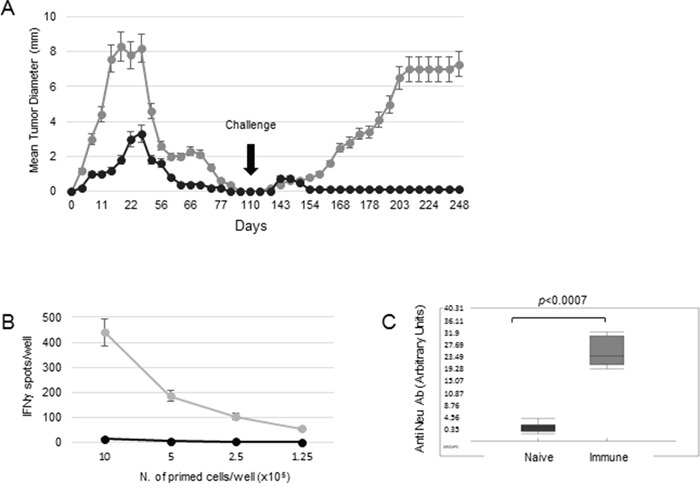
Immunization strategies against a HER-2 expressing transplantable tumor **A.** 129Sv mice were injected s.c. with 5×10^5^ (black circles) or 5×10^6^ (grey circles) of live 676-1-25 tumor cells. Tumor-free mice were re-challenged with 3×10^6^ live 676-1-25 cells 110 days later. Plots represent the mean tumor diameter per 6 mice per group ±SD. **B.** Splenocytes isolated from naïve mice (black) and immune mice vaccinated with 5×10^5^ live HER-2 tumor cells (grey) were plated in the indicated numbers and tested for IFN-γ production in the presence of the HER-2-specific 676-1-25 cell lysate, in an ELISPOT assay. The average value ±SD obtained from three independent experiments is shown. **C.** Box plot showing the levels of anti-HER-2 antibodies, detected by FACS analysis in the serum of vaccinated (grey) or naïve (black) 129Sv mice, collected 14 days post vaccination, measured for 6 samples per group. Black line: Median, Box: 25th to 75th percentile, whiskers:10th to 90th percentile. *p*< 0.001 (Mann Whitney).

The 676-1-25 cell line was used as a model of transplantable tumor for the design of different vaccination strategies against HER-2^+^ tumors. Preliminary experiments showed that mice receiving 676-1-25 tumor cell lysate as vaccine experienced a significant protection against live tumor cell challenge, that was more effective when the cell lysate was given in combination with CTX, administered one day before the vaccine (Supplementary Figure S2). However, upon a second tumor challenge (140 days after the first), all vaccinated mice developed tumors (Supplementary Figure S2), indicating that cell lysate immunization was ineffective in inducing a long-lasting anti-tumor immunity. As an alternative approach, mice were immunized with two doses (5×10^5^ and 5×10^6^) of live 676-1-25 tumor cells. As shown in Figure 1A, in both groups tumors were rejected in 100% of mice by 100 days from tumor injection. However, after a second injection with live 676-1-25 cells, only mice previously receiving 5×10^5^ live cells as vaccine experienced the complete protection from tumor challenge (Figure 1A). We conclude that vaccination with 5×10^5^ live cells represents a good strategy in protecting naïve mice against the challenge with HER-2^+^ tumor cells.

In order to investigate the correlates of protection of mice receiving 676-1-25 cells as live vaccine, we analyzed the immune phenotype and activation state of splenocytes isolated from mice vaccinated with 5×10^5^ live 676-1-25 tumor cells. Splenocytes isolated from vaccinated, but not from naïve mice, displayed strong IFN-γ production in response to HER-2-specific antigenic stimulus in a dose-dependent fashion (Figure 1B). The rejection of 676-1-25 tumor cells by vaccinated mice was also associated with a significant anti-Neu antibody response, as detected in mice sera on day 14 post injection (*p*<0.0007 Mann Whitney vs naïve mice) (Figure 1C).

The ensemble of these results indicated that injection of 5×10^5^ HER-2^+^ cells in syngeneic 129Sv stimulated an immune response against HER-2^+^ tumors that was protective against a subsequent challenge. This observation prompted us to test mice receiving this immunization regimen as T lymphocyte donors in protocols of adoptive cell transfer (ACT) to treat spontaneously arising syngeneic tumors.

### Therapeutic efficacy of chemo-immunotherapy

**Figure 2:**
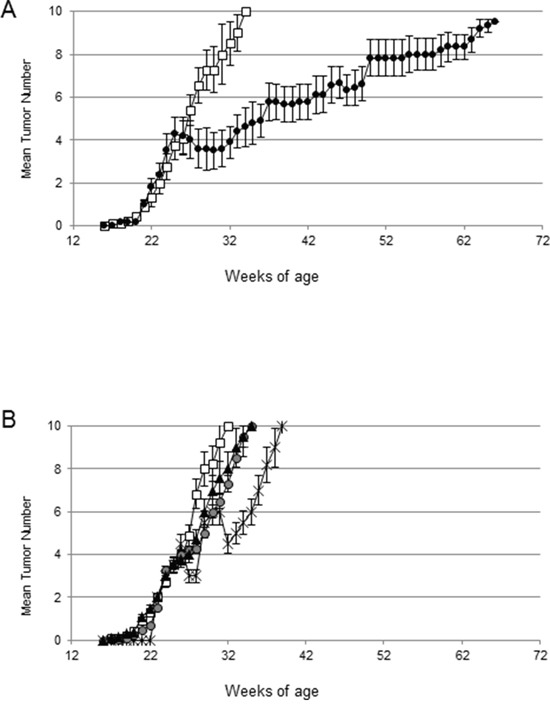
Efficacy of chemo-immunotherapy on the cure of large mammary tumors in HER-2/neu transgenic mice **A.** 129Sv-NeuT mice (10 mice per group), bearing established tumors on an average of 4/10 mammary glands, were left untreated (white boxes) or received chemo-immunotherapy (black circles), consisting of CTX (i.p.) plus splenocytes (i.v.) and immune serum (i.p.) from donor HER-2-vaccinated mice. Plot shows tumor multiplicity, calculated as the cumulative number of incident individual tumors/total number of mice, reported as mean ± SD. **B.** 129Sv-NeuT mice (10 mice per group), bearing established tumors on an average of 4/10 mammary glands were treated with ACT +IS (black triangles), CTX+ immune serum (asterisks), immune serum alone (grey circles), or left untreated (white boxes). Plot shows the mean tumor multiplicity ± SD. Data are representative of at least 2 independent experiments.

We tested the anti-tumor effect of the combination of CTX, immune serum (IS) and adoptive cell transfer of immune splenocytes from HER-2-vaccinated mice in tumor-bearing HER-2/neu transgenic mice (ACT/IS). We then selected 26 weeks-old mice, bearing established mammary tumors in an average of 4/10 mammary glands. The results of our experiments, reported in Figure 2, show that the combined chemo-immunotherapy regimen exerted a significant effect in HER-2/neu mice, inducing a noteworthy drop of tumor multiplicity starting immediately after treatment and peaking 4-5 weeks later (Figure 2A). Of note, out of the 4/10 pre-existing lesions per mouse detected at the time of CTX + ACT/IS therapy, an average of two tumors per mouse underwent regression, not always simultaneously (thus explaining the smaller drop visible in Figure 2A) but during the course of 6-7 weeks post treatment.

After that time, tumor multiplicity increased, albeit at a much lower rate as compared to control untreated mice. In fact, mice receiving the combined chemo-immunotherapy developed mammary carcinomas in all mammary glands with a median latency of 72 weeks, significantly longer than the median latency of 32 weeks displayed by untreated 129Sv-NeuT mice (Figure 2A). Of note, administration of IS, alone or in combination with ACT without CTX pre-conditioning, did not produce significant reduction of tumor growth in HER-2/neu mice, while mice receiving CTX + IS experienced a partial and very brief tumor reduction (Figure 2B). No effect could be ascribed to the direct effect of CTX, ACT or their combination to tumor-bearing HER-2/neu mice (Supplementary Figure S3).

**Figure 3:**
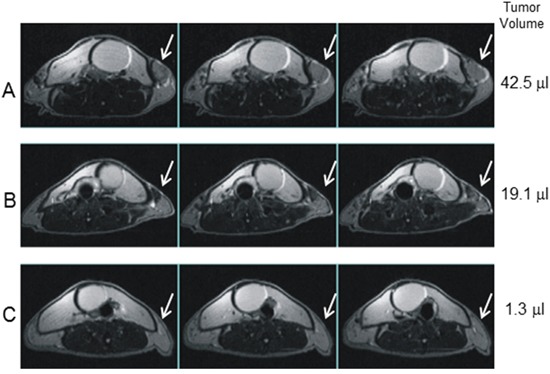
Magnetic resonance imaging of CTX+ACT/IS-treated tumor Multislice T2-weighted MRI (TR/TE=3000/70 ms) of the abdomen of a tumor-bearing HER-2/neu mouse, taken at day 1 **(A)** 16 **(B)** and 45 **(C)** after CTX+ACT/IS treatment, document treatment-induced reduction in tumour volume (white arrows). The shrinkage of the inguinal tumor, taken as representative of many masses undergoing treatment-induced disappearance, is visible soon after therapy (B) and it is completely regressed on day 45 (C).

Imaging analysis performed by multislice T2-weighted MRI at different time points after CTX + ACT/IS was used to monitor chemo-immunotherapy-induced changes in tumor size. Figure 3 shows serial slice images performed on day 1 (A), day 16 (B) and day 45 (C) post CTX + ACT/IS documenting the progressive shrinkage followed by the complete disappearance of one mammary carcinoma representative of the tumors undergoing regression in response to the therapeutic treatment.

**Figure 4:**
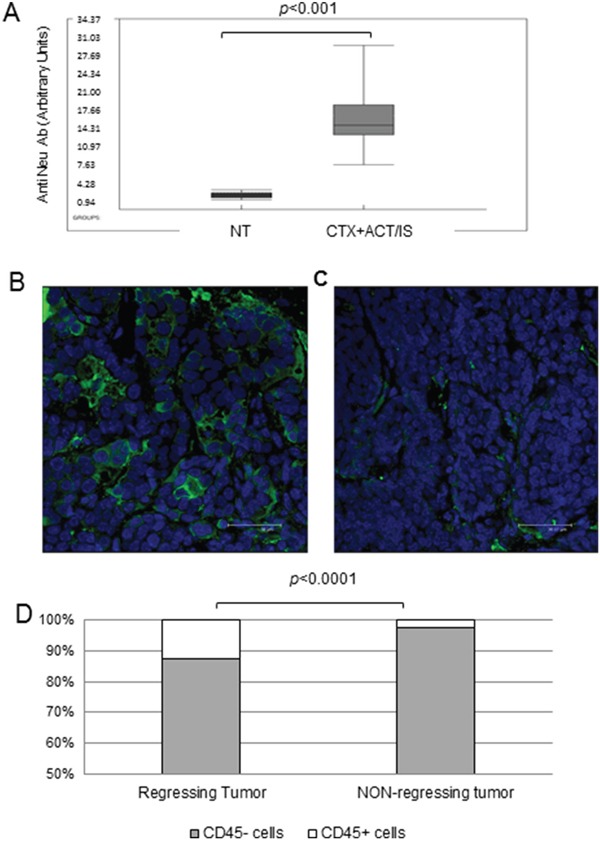
Immune correlates of CTX+ACT/IS efficacy on tumor-bearing transgenic HER-2/neu mice **(A)** The box plot shows the levels of anti-HER-2 antibodies, detected by FACS analysis, in the serum of tumor-bearing untreated or treated 129Sv-NeuT mice 20 weeks after CTX+ACT/IS, measured for 8 samples per group. Black line: Median, Box: 25th to 75th percentile, whiskers:10th to 90th percentile. *p*< 0.001 (Mann Whitney). Immunofluorescence staining with the pan leukocyte marker CD45 of a representative CTX+ACT/IS-treated **(B)** and an untreated tumor **(C)** taken 14 days post therapy. The images document the higher frequency of tumor infiltrating leukocytes (green fluorescence) in lesions undergoing regression. Bars correspond to 38 μm. **(D)** The histogram shows the incidence of CD45^+^ on the totality of cells, assessed according to the total number of DAPI-stained nuclei in each microscopy field. The average *p* value, calculated on 10 fields per condition, in regressing versus resilient tumors (n=3) is 0.0001 (Mann Withney).

In the attempt of identifying the immune correlates of chemo-immunotherapy efficacy, we analyzed the humoral immune response against HER-2 antigen in transgenic mice receiving CTX + ACT/IS therapy, that could account for long-term resistance to spontaneous tumor development. Overall, the level of anti-HER-2 antibodies detected in the serum of mice previously treated with CTX + ACT/IS experiencing tumor remission was significantly higher (*p*<0.001 Mann Whitney) than the amount found in tumor-bearing untreated mice (Figure 4A). Interestingly, when we matched serological and tumor monitoring data in groups of mice clustered according to CTX + ACT/IS efficacy (Supplementary Figure S4), we found that the amount of anti-Neu antibodies was highest in the group of mice experiencing a complete and long-lasting response (group 1), while anti-Neu Ab levels were progressively lower in mice showing relapses or partial response (groups 2 and 3).

To further determine the involvement of anti-tumor immune response in the shrinkage and subsequent disappearance of tumor masses observed in mice undergoing chemo-immunotherapy, confocal microscopy analysis was performed on FFPE sections prepared from regressing tumors isolated from CTX + ACT/IS-treated mice mice 14 days post injection (Figure 4B), and from non-regressing tumors explanted from untreated mice (Figure 4C). Staining with the pan-leukocyte marker CD45 showed significant levels of tumor infiltrating leukocytes in regressing CTX + ACT/IS-treated tumors but not in non-regressing tumors (Figure 4C). Of note, CD45^+^ cells in regressing tumors represented the 12% of total cells, while resilient tumors showed only a 2% of leukocyte infiltrate (*p*<0.0001 Mann Whitney) (Figure 4D).

### Prophylactic efficacy of chemo-immunotherapy

**Figure 5:**
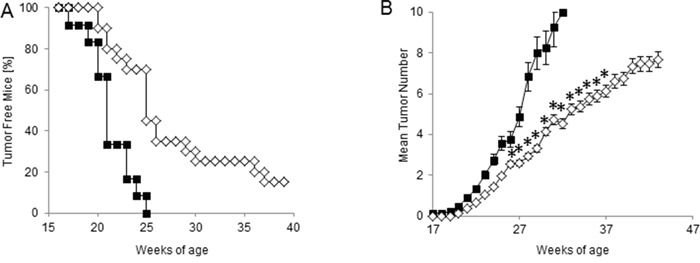
Effect of CTX+ACT/IS prophylactic vaccination on the spontaneous growth of mammary tumors in tumor-free HER-2/neu transgenic mice Tumor-free 129Sv-NeuT mice (10 mice per group) were left untreated (filled boxes) or immunized with chemo-immunotherapy (empty diamond), consisting of CTX+ACT/IS. **A.** Tumor incidence, expressed as the number of mice with at least one progressively growing tumor >1mm in diameter. **B.** Tumor multiplicity, calculated as the cumulative number of incident individual tumors/total number of mice, is reported as mean ± SD, for 10 mice per group. Data are representative of at least 2 independent experiments. (*p<0.05 Mann Whitney test).

In order to test the efficacy of our chemo-immunotherapy strategy in hampering the spontaneous carcinogenesis in HER-2/neu transgenic mice, protection experiments were performed. Thus, tumor-free 129Sv-NeuT mice received CTX and ACT/IS at 17 weeks of age, when no tumor was palpable in any of the mammary glands. The experimental end points considered were the age at the onset of the first mammary carcinoma, and the total average number of tumors per mouse over time. The results of manual inspections showed a significant delay in tumor appearance in HER-2/Neu mice undergoing prophylactic CTX + ACT/IS treatment with respect to untreated mice, with 20% of mice being still tumor-free at 40 weeks of age (Figure 5A). Moreover, CTX + ACT/IS significantly decreased the number of mammary glands with palpable tumor as compared to controls (*p*<0.05 Mann Whitney test) (Figure 5B). Notably, the median time interval from tumor onset to death was 20 weeks for untreated control mice, and over 30 weeks for CTX + ACT/IS-vaccinated mice, indicating the remarkable efficacy of the chemo-immunotherapy prophylactic regimen in prolonging the overall survival in HER-2/neu mice. None of the single control treatments (CTX, ACT and IS alone) had any effect on lesion appearance and tumor multiplicity throughout mice life (*data not shown*).

## DISCUSSION

The requirement for a suitable animal model to address the open issues of chemo-immunotherapy often collides with the limitations of transplantable murine cancer cell lines, not faithfully recapitulating human cancer [22]. Mice transgenic for the rat Neu proto-oncogene, expressed under the control of a mammary-specific promoter, naturally develop focal mammary adenocarcinomas and represent a reliable model of immune tolerance resulting in spontaneous mammary carcinogenesis [23]. The double property of HER-2, being at the same time a self-antigen and a constitutively-expressed oncogene, makes the prevention and/or cure of HER-2 spontaneous mammary tumors particularly stimulating and challenging for immunotherapy.

We here show that the anti-tumor immunological memory, induced by vaccination with live HER-2^+^ tumor cells in 129Sv naïve recipients mice, can be transferred to transgenic tumor-bearing HER-2/neu mice, where the combination of CTX and ACT/IS hampers carcinogenesis and causes the regression of large mammary tumor lesions.

P185-expressing tumor cells, when injected s.c. in low numbers (5×10^5^ cells) in wild type mice, induced the development of self-limiting tumor masses, undergoing spontaneous rejection in 100% of mice within four months. Although vaccination with both live or lysed HER-2^+^ tumor cells protected mice from tumor challenge, only the sub-tumorigenic dose of live cells induced a durable protection from subsequent challenge, revealing that an effective anti-tumor immunological memory was elicited. Among different possible mechanisms, we can hypotesize that the low numbers of live tumor cells multiply at slow rate when injected *in vivo*, generating large amounts of immunizing antigens to mediate tumor rejection before sizeable masses develop, thus reaching a favorable balance between immunogenicity and tumorigenicity. In fact, mice receiving this vaccine exhibited activated-anti HER-2 T lymphocytes, and held anti-p185/HER-2 antibodies in the sera. These observations prompted us to employ these vaccinated mice as immune donors in a chemo-immunotherapy approach, to be tested in tumor-bearing HER-2/neu transgenic mice.

Of note, transgenic 129Sv-NeuT mice bearing large mammary tumors exhibited a remarkable response to the combined treatment with CTX plus immune cells and sera from vaccinated mice, as shown by treatment-induced dramatic reduction in the number of pre-existent tumor nodules. The finding that ACT or IS alone were ineffective in restricting tumor growth in 129Sv-NeuT mice suggest that CTX is necessary to overcome anti-tumor immune tolerance and to induce an antigen-specific effective immune response in these mice.

Many studies published by our group and others demonstrated that some chemotherapeutic agents, administered at non-myeloablative doses shortly before immunotherapy, enhance the efficacy of adoptive transfer of antigen-specific lymphocytes and tumor vaccines [8]. As mentioned above, the improved efficacy of a cell-based vaccine in reducing HER-2^+^ spontaneous tumor sizes when combined with chemotherapy has been observed. [16] However, to the best of our knowledge, the complete eradication of tumor masses described in this study, as detected by manual inspection and confirmed by MRI analysis in transgenic mice undergoing chemo-immunotherapy, has never been reported before.

The durable regression of large tumor lesions represents a big trial for cancer therapies. In fact, treatments that prove effective in stimulating the immune-mediated rejection of small lesions are often unsuccessful against well-established solid tumors, where other factors can support tumor escape, including the development of a tumor-promoting stromal microenvironment [22, 24]. This phenomenon is particularly evident in transplantable tumor models, where early or superficially spreading tumors have more chances than large tumors to be rejected. In this light, our evidence that spontaneously arising large mammary tumors completely regress following chemo-immunotherapy represents a relevant achievement. Moreover, our model of tumor regression in autochthonous mice provides a good chance to further characterize the key elements for an effective chemo-immunotherapy approach.

In our study, one single administration of CTX + ACT/IS, tested as a prophylactic strategy in tumor-free mice, caused a significant delay in the appearance of spontaneous tumor masses development with respect to untreated HER-2/neu mice. It is worth mentioning that other vaccines reported to be effective in blocking or hindering mammary carcinogenesis, required lifelong administration to ensure a complete and durable protection, whereas shorter and/or delayed protocols eventually left mice exposed to tumor onset [25]. Our results indicate that pre-conditioning with CTX created the right conditions for the transferred immune cells to brake the intrinsic tolerance against the self onco-antigen HER-2. Although the tumor-promoting effect of HER-2 overexpression eventually caused tumor escape and lesions outbreak, the protective effect we achieved with one single vaccination was remarkably durable when compared with other vaccination schedules [26]. Nevertheless, our results deserve further investigation to achieve more effective and long-lasting protection. Interestingly, the observation that CTX + ACT/IS exert a prophylactic effect in tumor-free 129Sv-NeuT mice also suggests that the therapeutic efficacy of this regimen observed in tumor-bearing mice may occur not only through a direct cytotoxic effect on the tumor mass, but also by the stimulation of an effective antitumor immune response.

In support of this concept, the combined chemo-immunotherapy approach, with respect to single treatments, promoted a greater systemic immune response reflected by higher serum levels of anti p185 antibodies and a stronger local immune response reflected by enhanced tumor-infiltrating lymphocyte populations.

Antitumor humoral immune response has been often associated with the efficacy of vaccination strategies capable of controlling spontaneous carcinogenesis in HER-2/neu transgenic mice in prophylactic regimens [15]. It has been described that serum anti-p185neu antibodies may cause a functional block of p185neu receptor function, down-regulating its expression on the cell membrane [27], and impeding the formation of homo or heterodimers that transduce proliferative signals to the cells [27, 28]. In our experiments, we observed a suggestive relationship between the amount of anti-HER-2 serum antibodies and the extent of therapeutic response in tumor-bearing mice undergoing CTX + ACT/IS. Interestingly, IS administered alone did not produce any effect on pre-existing tumor masses, suggesting that, although the transfer of tumor-specific immunoglobulins was an important component for therapeutic success, CTX + ACT/IS-induced tumor regression occurred through more complex mechanisms, involving both humoral and cellular immunity, and requiring CTX pre-conditioning.

Tumor infiltrating lymphocytes can have an antitumor but also immunosuppressive role, and the efficacy of immunotherapy may rely on the possibility to shift the balance from immune suppression to antitumor activity at the microenvironment level. In particular, CTX may act through the depletion and/or functional suppression of regulatory T cells [29]. In our study, the presence of infiltrating CD45^+^ cells in regressing tumor masses undergoing CTX + ACT/IS but not in resilient tumors receiving single treatments, strongly suggests that the immune-enhancing effect of CTX pretreatment followed by ACT/IS may be explained, at least in part, by an increase in the presence of antigen-specific T cells at the tumor site. Whether the observed tumor rejection is also mediated by a more efficient recruitment/stimulation of antigen-presenting cells, and the enhancement of innate immune responses occurring in response to CTX needs further investigation. It is tempting to speculate that the efficacy of the combined treatment in tumor-bearing mice relies on the danger signals generated in cancer cells by CTX, eventually stimulating a more efficient anti-cancer immune response. In fact, it has been reported that CTX, among other chemotherapeutic agents, can induce an “immunogenic” type of tumor cell death that stimulates tumor-specific immunity by potentiating tumor infiltration, engulfment of tumor apoptotic material, and CD8 T-cell cross-priming [11, 30].

Overall, our data support the immune-modulatory role of chemotherapy in overcoming cancer immune tolerance when administered at lymphodepleting non-myeloablative doses shortly before antigen-specific immunotherapy. In our view, HER-2/neu transgenic mice experiencing the regression of established spontaneous tumors following combined CTX + ACT/IS treatment represent an invaluable animal model to better explore the requirements of the host for an effective anticancer chemo-immunotherapy. Our results are in support of a potential therapeutic for cancer patients or for individuals genetically prone to tumor development, with the aim of improving their spontaneous antitumor immune response.

## MATERIALS AND METHODS

### Mice

Healthy young (8–16 weeks old) female 129Sv mice were purchased from Charles River Laboratories (Calco, Italy). A colony of 129Sv-NeuT transgenic mice were generated and bred in the animal facility of the Haemathology and molecular Oncology Department at the Istituto Superiore di Sanità (Rome, Italy). Pure 129Sv-NeuT transgenic mice were obtained by at least 12 backcrosses of BALB-NeuT transgenic males (kindly provided by Dr. Guido Forni) with female 129Sv mice. The BALB-NeuT strain originated from a transgenic CD1 random-bred breeder male mouse (no. 1330) carrying the mutated rat HER-2/neu oncogene driven by the MMTV promoter [13]. The mutated gene encodes a single point mutation that replaces the valine residue at position 664 in the transmembrane (TM) domain of p185/neu with glutamic acid. This mutation promotes p185/NEU homo- and heterodimerization and transforms the HER-2 proto-oncogene into a dominant transforming oncogene. 129Sv-NeuT Virgin females develop spontaneous mammary carcinomas with a mean latency time of about 20 weeks. Mice were maintained under strict inbreeding conditions. The presence of the rat HER-2 transgene was routinely checked by polymerase chain reaction (PCR) on tail DNA using primers hybridizing to vector (5-ATCGGTGATGTCGGCGATAT-3) and to MMTV sequences (5-GTAACACAGGCAGATGTAGG-3). The mammary glands of all 129Sv-NeuT transgenic virgin females mice were inspected once a week for tumor monitoring. Individual neoplastic masses were measured with calipers in 2 perpendicular diameters and the mean value was recorded. Progressively growing masses with mean diameter >1 mm were regarded as tumors. Mice bearing tumor masses exceeding 30mm mean diameter were euthanized. Tumor multiplicity was calculated as the cumulative number of incident individual tumors/total number of mice and is reported as mean ± standard deviation (SD). Mice were housed at the ISS animal facility. Investigation using animals has been conducted in accordance with the ethical standards and according to the Italian DL 26/14, enforcing the European Directive 2010/63 EEC on Laboratory Animal Welfare.

### Tumor cell lines

A tumor cell line isolated was isolated from a spontaneous HER-2^+^ mammary tumor developed in 129Sv-NeuT transgenic virgin female, stabilized *in vitro* and named 676-1-25 (Supplementary Materials and Methods). N202.1A and N202.1E [31] are cloned cell lines derived from a FVB mice (H-2q) transgenic for the r-Her-2/neu proto-oncogene (FVB-NeuN). N202.1A cells express high levels of surface HER-2/neu protein, while N202.1E cells are the HER-2^−^ counterpart. Both cell lines were obtained as a gift from Dr. Claudia Curcio, tested by FACS for HER-2 expression and used within six months for serum anti HER-2 antibody analysis. All cells were cultured in RPMI 1640 supplemented with 10% FBS.

### Chemoimmunotherapy

For vaccination experiments, HER-2^+^ tumor cells lysate was prepared by three rounds of freezing/thawing of 676-1-25 tumor cells. Mice were immunized by s.c. injection of 0.1ml of cell lysate, corresponding to 5×10^6^ live cells, or with different doses of 676-1-25 live cells. Cyclophosphamide (CTX, Sigma) was administered intraperitoneally at 100mg/kg one day before immunization. Mice were then challenged with 3×10^6^ 676-1-25 live cells and monitored for tumor development.

For the preparation of immune cells and sera, 129Sv donor mice were immunized with three s.c. inocula, at a time interval of 2 weeks, with 5×10^5^ 676-1-25 cells, and an extra boost one week before adoptive cell transfer. At the time of chemo-immunotherapy, donor mice were killed, and spleens and blood were removed aseptically. Single-cell suspension of spleen cells were prepared after erythrocytes lysis by 3-min incubation at room temperature in 0.16M Tris–buffered NH_4_Cl. Cells were washed in complete medium, passed through a cell strainer (Falcon 2350; Becton Dickinson, Inc.) and resuspended to 2.5×10^8^ viable cells/ml (determined by Trypan blue exclusion) in RPMI 1640 medium with 2% FBS. Immune serum was prepared as follows: peripheral blood was collected from donor mice in a sterile tube, left at 37°C for 30 minutes and then centrifuged at 4°C at 2.000 rpm for 20 minutes and stored at −20°C.

For chemo-immunotherapy experiments, 129Sv-NeuT mice bearing established tumors on an average of 4/10 mammary glands (at around 26 weeks of age for therapeutic approaches) or still tumor-free (17 weeks of age for prophylactic approaches), were injected i.p. with 100 mg/kg CTX. Five hours later, recipient mice were injected intravenously with 0.4 ml of a suspension of 10^8^ spleen cells freshly prepared from donor mice. One day later, mice received i.p. injection of 0.2ml of immune serum diluted 1:2 in saline. Recipient mice were then inspected once a week for tumor growth.

### Flow cytometry

The product of the transgene, rat HER-2, was detected in the 676-1-25 cell line using anti-c-ErbB2/c-Neu (Ab-4) antibody (clone 7.16.4) mouse monoclonal antibody (Calbiochem). Briefly, cells were stained with the anti-Neu primary antibody followed by a fluorescein-conjugated rabbit anti-mouse IgG antibody (DAKO). Cells were then washed and resuspended in PBS containing 1 μg/ml of propidium iodide to gate out dead cells, and analyzed by flow cytometry in a FACS Calibur instrument (Becton Dickinson). For anti-Neu antibody response evaluation, whole blood samples were obtained at several time points after mice immunization or chemo-immunotherapy, serum was separated and stored at −20°C. N202.1A cells (HER-2^+^) and N202.1E cells (HER-2^−^) were used to quantify anti-Neu antibodies as previously described [32]. Briefly, 2×10^5^ cells were incubated with test and control sera diluted 1:10 in 1% FBS in PBS at 4°C for 1 h. Cells were washed and incubated with FITC-labeled rabbit anti-mouse immunoglobulin antibody (DAKO) and mean fluorescence intensity was measured by flow cytometry. The non-specific anti HER-2 signal, obtained in samples incubated with N202.1E cells, was used as background and subtracted from the specific labeling in sera incubated with N202.1A cells. Stained cells were analysed in a FACS Calibur instrument (Becton Dickinson). Anti-Neu Arbitrary Units were calculated as [% of positive cells with test serum – % of positive cells with control serum].

### ELISPOT

For IFN-γ enzyme-linked immunospot assay, nitrocellulose-bottomed 96-well plates (MultiScreen HTS-IP, Millipore) were coated overnight at 4°C with anti-IFN-γ mAb (AN18 Mabtech). Plates were blocked with 10% FBS-containing medium for at least 30 min at room temperature. Erythrocyte-depleted spleen cells from immunized or naive mice were plated in triplicates at four different cell concentrations (1×10^6^ to 1.25×10^5^ c/well) including HER-2^+^ cell line lysate as antigen (5:1 responder to stimulator ratio). Plates were then incubated overnight at 37°C, washed five times and incubated with biotinylated anti-IFN-γmAb (R4-6A2-biotinMabtech) for 2 hours at room temperature. After washing, streptavidin-enzyme conjugate (Mabtech) was added to each well and incubated for 1 hour at room temperature, followed by 5-bromo-4-chloro-3-indolyl phosphate/nitroblue tetrazolium (Sigma). The reaction was terminated on the appearance of dark spots by washing the plates with tap water. The spots were counted using the 4-Plate ELISPOT Reader V2.1 (Aelvis GmbH).

### Magnetic resonance imaging

Magnetic Resonance Imaging (MRI) analysis was conducted at 4.7 T on a Varian/Agilent Inova horizontal bore system (Agilent) using a volume coil as transmitter and a surface coil as receiver (RAPID Biomedical). Animal anaesthesia was induced with isofluran 2.5% in O2, 1 L/min, and reduced to 1.5-2.0% in O2, 1 L/min, once the animal became unresponsive to paw pinch. Multislice coronal and axial anatomical T1-weighted and T2-weighted MRI were acquired by using the following parameters: TR/TE = 3000/70 ms for T2-weighted and TR/TE = 660/18 ms for T1-weighted images, 4 transients, 37 slices, FOV = 30 × 30 mm2, matrix 256 × 128, thickness = 0.8 mm. MRI of tumors was carried out at day 1, 16 and 45 days after treatment, during tumour growth progression.

### Immunofluorescence and confocal microscopy

Immunofluorescent labelling was performed on Formalin Fixed Paraffin Embedded (IF-FFPE) tissue sections. Slides (5 μm thick) were deparaffinized, re-hydrated through graded alcohols and subjected to a Heat-induced Epitope Retrieval step by Citrate pH6 (Novus) for 3×3min in MW. Sections were washed with PBS-T and blocked in PBS-BSA 3% for 30 min at 37°C. Primary antibody (anti-mouse CD45, Santa Cruz) was added in PBS-BSA 3% and incubated 30 minutes at 37°C. After washing, sections were incubated for 30 minutes at 37°C with the secondary antibody anti-mouse AlexaFluor 488 (Invitrogen) plus DAPI (Life Technologies). Sections were mounted in Vectashield anti-fade mounting medium (Vector Laboratories) and fluorescence images were captured by means of Leica TCS SP2 microscope (Leica Microsystems). The percentage of positive cells was calculated from 10 random fields.

## SUPPLEMENTARY DATA AND FIGURES


