# Supplementary material for: Chemo-immunotherapy induces tumor regression in a mouse model of spontaneous mammary carcinogenesis
Source: Oncotarget. 2016 Jul 28;7(37):59754–65. doi: 10.18632/oncotarget.10880 (PMC5312346; doi:10.18632/oncotarget.10880)
Supplement: Supplementary file 1 [file oncotarget-07-59754-s001.pdf]

Chemo-immunotherapy induces tumor regression in a mouse model of spontaneous mammary carcinogenesis

SUPPLEMENTARY DATA

HER2$^+$ mammary carcinoma cell line generation and characterization

Tumor samples isolated from 129Sv-NeuT mice were freed from hemorrhagic and necrotic parts, washed in phosphate-buffered saline (PBS), finely minced with scissors and digested in a standard tissue culture grade trypsin-EDTA solution (0.5 mg/ml trypsin, 0.2 mg/ml EDTA, Life Technologies) at 37°C for 15 min; dissociated cells were washed twice in PBS and counted in a hemocytometer. Cells were seeded in tissue culture flasks in RPMI 1640 supplemented with 10% FBS (Life Technologies) and incubated at 37°C in a humidified 5% CO_2atmosphere. Cultures were periodically washed briefly (1–2 min) with trypsin-EDTA to detach contaminating fibroblasts without damage to epithelial areas. When the epithelial monolayer reached confluency, usually 2–5 months after plating, cells were subcultured at low split ratios (usually 1:2). Established cell line named 676-1-25 was routinely subcultured twice weekly at 1:4–1:8 split ratios. For *in vitro* growth curve, cells were cultured in 24 well plates in triplicates at $2x10^4$ cells per well, then harvested and counted at various times. For *in vivo* growth, healthy female 129Sv mice were used for the analysis of tumorigenicity of cultured cells. Briefly, tumors were induced by subcutaneous (s.c.) injection of varying amounts of 676-1-25 tumor cells as a single-cell suspension in PBS (0.2 ml/mouse). Tumor incidence and growth were evaluated twice weekly. Neoplastic masses were measured with caliper and the mean tumor diameter was calculated in 2 perpendicular major diameters.

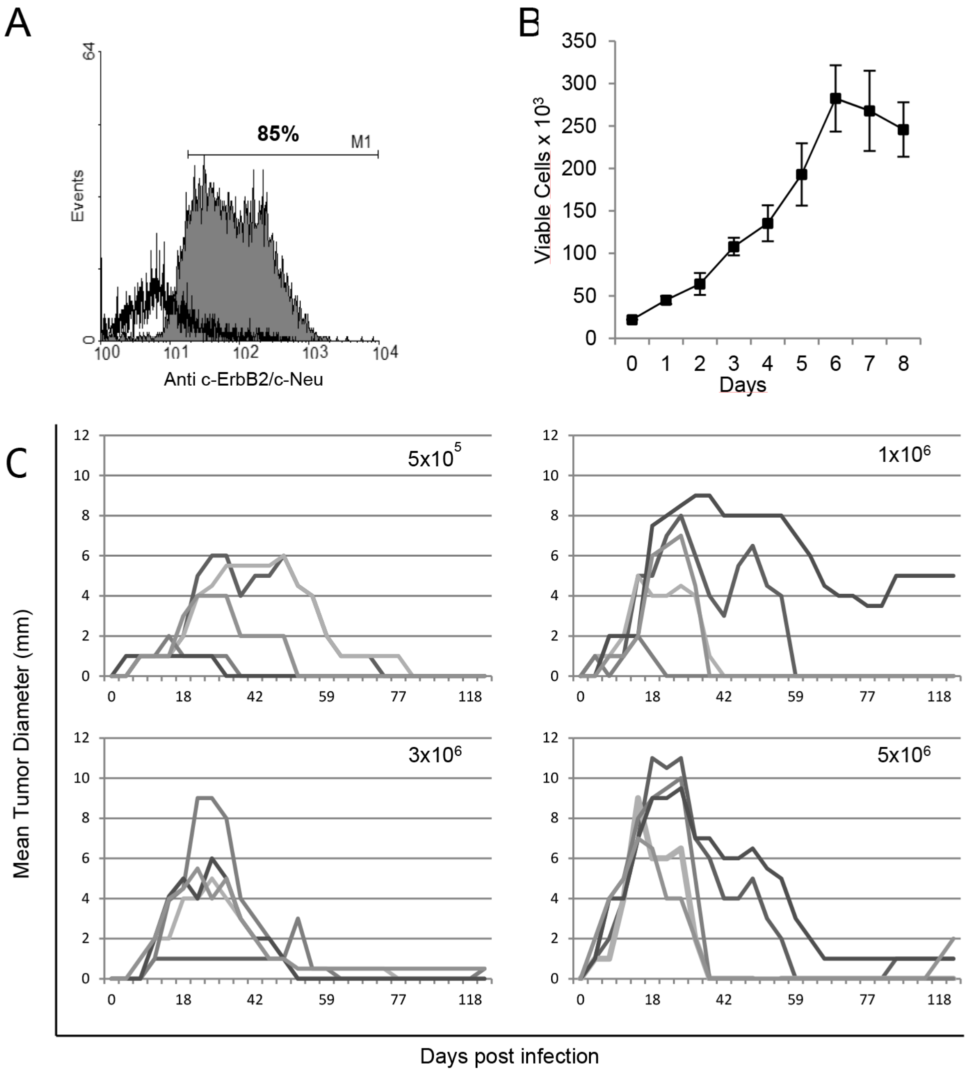

Supplementary Figure S1: Characterization and growth properties of the HER2$^+$ mammary carcinoma 676-1-25 cell line. The 676-1-25 cell line was isolated and stabilized *in vitro* from a mammary tumor spontaneously arisen in 129Sv HER-2/neu$^+$ transgenic mouse. **A.** Cell surface expression of HER2 was analyzed by flow cytometry using non-permeabilized cells stained with anti-c-ErbB2/c-Neu antibody. Empty histogram represent isotype control antibody-stained cells. **B.** *In vitro* growth curve of 676-1-25 was determined using trypan blue exclusion assay in triplicates. The graph shows the average number of cells ± SD. **C.** *In vivo* growth curve of 676-1-25 cells, injected s.c. in 129Sv mice at the indicated doses. Tumor diameters are shown for each of 5 mice per group. Data are representative of at least 2 independent experiments.

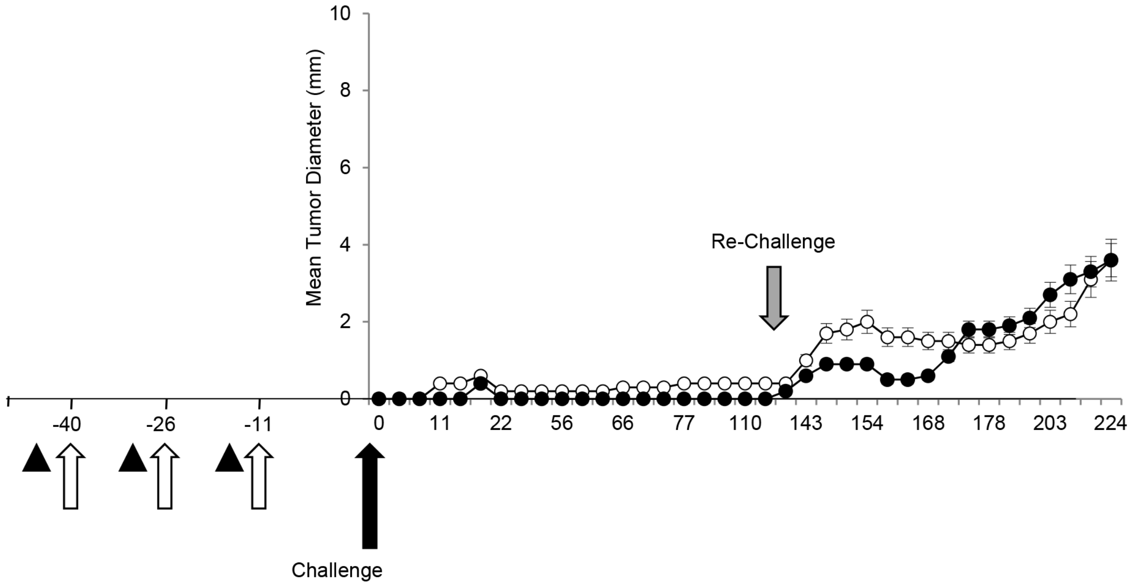

Supplementary Figure S2: Vaccination strategies using 676-1-25-induced tumors. 129Sv mice (5 per group) were vaccinated, at two weeks interval (white arrows), with 676-1-25 tumor cell lysate equivalent to 5×10^6 cells. Tumor lysate was administered alone (open circles) or in combination with CTX plus ACT (filled circle). CTX (arrowheads) was injected 5 hours before tumor lysate. Vaccinated mice were then challenged with 3×10^6 live 676-1-25 cells. Mice that were tumor-free on day 140 underwent a second tumor challenge and monitored afterwards. Graph shows the mean tumor diameter per each group ±SD.

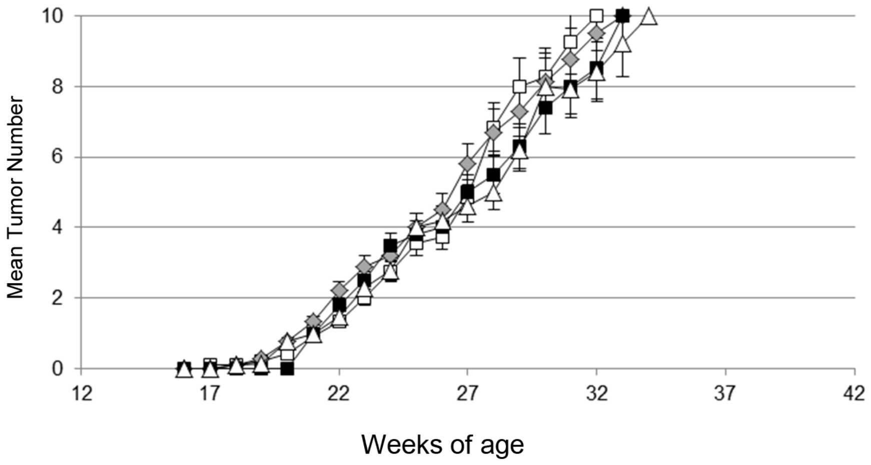

Supplementary Figure S3: Effect of CTX, ACT or their combination on spontaneous tumors established in HER-2/neu+ transgenic mice. 129Sv-NeuT mice (10 mice per group), bearing established tumors on an average of 4/10 mammary glands, were left untreated (white boxes) or received CTX i.p. (black boxes), ACT i.v. from donor HER-2-vaccinated mice (white triangles) and the combination of CTX + ACT (grey diamonds). Plot shows tumor multiplicity, calculated as the cumulative number of incident individual tumors/total number of mice, reported as mean ± SD. Data are representative of at least 2 independent experiments.

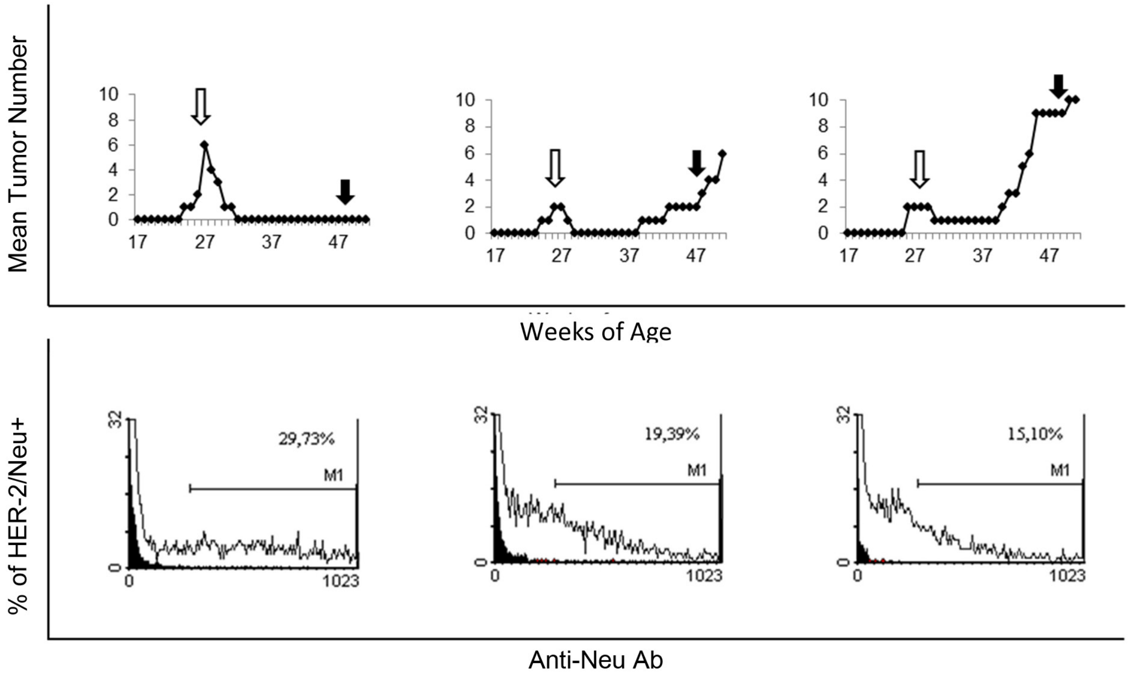

Supplementary Figure S4: Parallel between treatment-induced reduction of tumor masses A. and serum anti HER-2 antibodies B. in tumor bearing 129Sv-NeuT mice undergoing chemo-immunotherapy. 129Sv-NeuT mice received CTX+ACT/IS treatment (indicated by the white arrow) and were monitored for tumor multiplicity over time. Five months after treatment (black arrow), mice were sacrificed and blood was pooled for anti-Neu antibody response evaluation, performed as described in Materials and Methods. Panel B shows three different representative FACS profiles of N202A cells stained with mice sera containing HER2 antibodies, after subtraction of the non HER2-specific signal (shaded area), indicative of the amount of humoral anti-tumor immune response. Three different mice groups (4 mice each) have been selected and clustered for being representative of the different trends observed in the whole group of treated mice: complete and long-lasting regression (group 1, left): 29.73% of anti-HER-2 antibodies; complete regression followed by relapse (group 2, middle): 19,39% of anti-HER-2 antibodies; partial regression and relapse (group 3, right): 15.1% of anti-HER-2 antibodies.
